# Insight into the Assembly Properties and Functional Organisation of the Magnetotactic Bacterial Actin-like Homolog, MamK

**DOI:** 10.1371/journal.pone.0034189

**Published:** 2012-05-07

**Authors:** Sanjiv Sonkaria, Gloria Fuentes, Chandra Verma, Ram Narang, Varsha Khare, Anna Fischer, Damien Faivre

**Affiliations:** 1 Department of Biomaterials, Max Planck Institute of Colloids and Interfaces, Potsdam, Germany; 2 Bioinformatics Institute, A-STAR, Singapore, Singapore; 3 School of Biological Sciences, Nanyang Technological University, Singapore, Singapore; 4 Department of Biological Sciences, National University of Singapore, Singapore, Singapore; 5 Max Planck Institute of Molecular Plant Physiology, Bioinformatics Group, Potsdam, Germany; 6 Department of Colloid Chemistry, Max Planck Institute of Colloids and Interfaces, Potsdam, Germany; 7 Institute of Chemistry, Technical University Berlin, Berlin, Germany; University of Crete, Greece

## Abstract

Magnetotactic bacteria (MTB) synthesize magnetosomes, which are intracellular vesicles comprising a magnetic particle. A series of magnetosomes arrange themselves in chains to form a magnetic dipole that enables the cell to orient itself along the Earth’s magnetic field. MamK, an actin-like homolog of MreB has been identified as a central component in this organisation. Gene deletion, fluorescence microscopy and *in vitro* studies have yielded mechanistic differences in the filament assembly of MamK with other bacterial cytoskeletal proteins within the cell. With little or no information on the structural and behavioural characteristics of MamK outside the cell, the *mamK* gene from *Magnetospirillium gryphiswaldense* was cloned and expressed to better understand the differences in the cytoskeletal properties with its bacterial homologues MreB and acitin. Despite the low sequence identity shared between MamK and MreB (22%) and actin (18%), the behaviour of MamK monitored by light scattering broadly mirrored that of its bacterial cousin MreB primarily in terms of its pH, salt, divalent metal-ion and temperature dependency. The broad size variability of MamK filaments revealed by light scattering studies was supported by transmission electron microscopy (TEM) imaging. Filament morphology however, indicated that MamK conformed to linearly orientated filaments that appeared to be distinctly dissimilar compared to MreB suggesting functional differences between these homologues. The presence of a nucleotide binding domain common to actin-like proteins was demonstrated by its ability to function both as an ATPase and GTPase. Circular dichroism and structural homology modelling showed that MamK adopts a protein fold that is consistent with the ‘classical’ actin family architecture but with notable structural differences within the smaller domains, the active site region and the overall surface electrostatic potential.

## Introduction

The cytoskeleton is central to the organisation of a variety of molecular processes associated with cell shape maintenance, molecular trafficking and DNA segregation among other cellular functions [1], [2]. The boundary that restricted the superfamily of actin**-**like proteins exclusively to eukaryotic cells has in recent years been re**-**defined, with the discovery of filamentous proteins of prokaryotic origin. Among the most widely studied actin and tublin**-**like prokaryotic proteins include the DNA segregating protein ParM [3], FtsZ involved in cell division [4], [5] and MreB implicated in cell shape maintenance [6], [7], [8]. MamK is another example of an actin related homolog that uniquely functions in the alignment of magnetosomes found in magnetotactic bacteria [9], [10]–[12]. The low sequence identity shared among these proteins suggests inherent differences at the morphological and structural level. Such differences may be important in defining their unique cellular functions. It is however, not clear how variations in structure and homology might affect the general behavioural characteristics of the proteins.

MamK is a member of actin**-**like bacterial proteins but shows sufficient distant sequence homology with actin and other proteins of the same family to be classed as phylogenetically different [13]. MamK is among a number of actin-like proteins found in magnetotactic bacteria that include FtsZ [4], [5] and MreB which has been extensively studied in other bacterial systems [6], [7], [14]–[16]. MamK however, has perhaps evolved uniquely to self**-**sufficiently function in magnetosome organisation [11], [12]. MamK was shown to be involved in the optimal alignment of magnetosome chains in association with MamJ which is thought to act as a connector between MamK and magnetosomes [13]. This model was supported by gene deletion studies directly implicating *mamJ* in the organisation of the magnetosome chain which collapsed in its absence in the *M*. *gryphiswaldense MSR-1* strain. This finding contradicts the magnetosome arrangement observed for *Magnetococcus sp. MC-1* which retains its ability to form magnetosome chains even though the genus does not encode the *mamJ* gene [17]. The functional role of *mamJ* in this model therefore requires further investigation. The cytoskeletal framework of MamK is critically important in maintaining magnetosomes in straight chains to maximise the cellular dipole along the length of the chain and thus enabling magnetotaxis to occur optimally [18], [19]. Linear filaments were observed in *Magnetospirillium* cells even in areas where magnetosomes were absent [11] and it has been shown that recombinantly cloned MamK from *M. magnetotacticum* forms cytoskeletal structures and filament bundles when expressed *in vitro* [20]. The discovery of a second MamK**-**like protein in AMB**-**1 cells [21] represents an interesting development in the evolution of this protein. Despite a concerted effort, the precise role of MamK continues to remains elusive. Speculation that MamK may be involved in additional cellular activities [19] remains to be established conclusively. The question fundamentally centres on how MamK is able to maintain the integrity and dynamics of the magnetosome chain and how the process may be replicated *in vitro*. With little or no information relating to the assembly and structural properties of MamK, a deeper understanding of its actin**-**like behaviour outside the bacterial cell may help in defining some of the characteristics. The results of the study may also be usefully applied for the biomimetic organisation of magnetosomes and possibly other nanoparticles *in vitro.*

The work presented here demonstrates that MamK exhibits cytoskeletal behaviour typical of actin**-**like proteins using dynamic light scattering, circular dichroism and polymerization assays. The assembly properties investigated here in response to physico**-**chemical conditions influencing the monomer**-**polymer equilibrium was shown to strongly resemble those reported for MreB from *Bacillus subtilis* [14]. Computational models of three**-**dimensional homology modelling of MamK based on homology with actin and MreB lends further support to the remarkable structural similarity shared between these proteins. The modelling also predicts structural deviations and variability around the smaller domains and overall pattern of electrostatic potentials that may explain some of the differences that exist in the dynamics and morphology in these proteins as observed by TEM.

## Results

The *mamK* gene is encoded within the *mamAB* gene cluster that forms part of the magnetosome island in magnetotactic bacteria [22]. A homology**-**based search reveals a limited overlap in sequence identity (≤22%) with known actin**-**like proteins. The search pattern further shows that the active**-**site of MamK relates closely to that of the actin**-**like fold of MreB. MamK was cloned and studied as an isolated protein to address the functional relevance of MamK in terms of its biochemical and structural characteristics in comparison with actin and MreB.

### Purified MamK Shows a Partial Tendency to Spontaneously Polymerise *in vitro* in the Presence of Di-valent Metal Ions but in the Absence of ATP

The *mamK* gene was cloned and over-expressed in *E. coli* as a poly-six-histidine**-**tagged soluble protein. Concentration dependent elution of the protein with increasing imidazole was consistent with the presence of the tag (Figure S1). MamK was subsequently purified to homogeneity as a 42 KDa protein (Figure S1) identical to the His**-**tagged MamK protein isolated from *M. magnetotacticum* AMB-1 [20], [21]. In the presence of the elution buffer, MamK was completely soluble at 4°C evident from the lack of a visible pellet. Dialysis against polymerisation buffer (P.B) in the presence of Mg^2+^ ions followed by ultracentrifugation at 90,000 *g* resulted partly in the deposition of aggregates of MamK at 4°C. Furthermore, electrophoretic analysis of protein bands under native conditions after incubation at room temperature in the presence of divalent cations and in the absence of ATP revealed a partial tendency to form polymerised filaments (data not shown). These bear little or no ability to migrate through the gel (7.5% native polyacrylamide) probably due their shear hydrodynamic size, a characteristic also demonstrated by G**-**actin when compared under similar conditions.

### ATP Induced Polymerisation of MamK Shows Concentration and Time Dependent Increases in Filament Assembly

**Figure 1:**
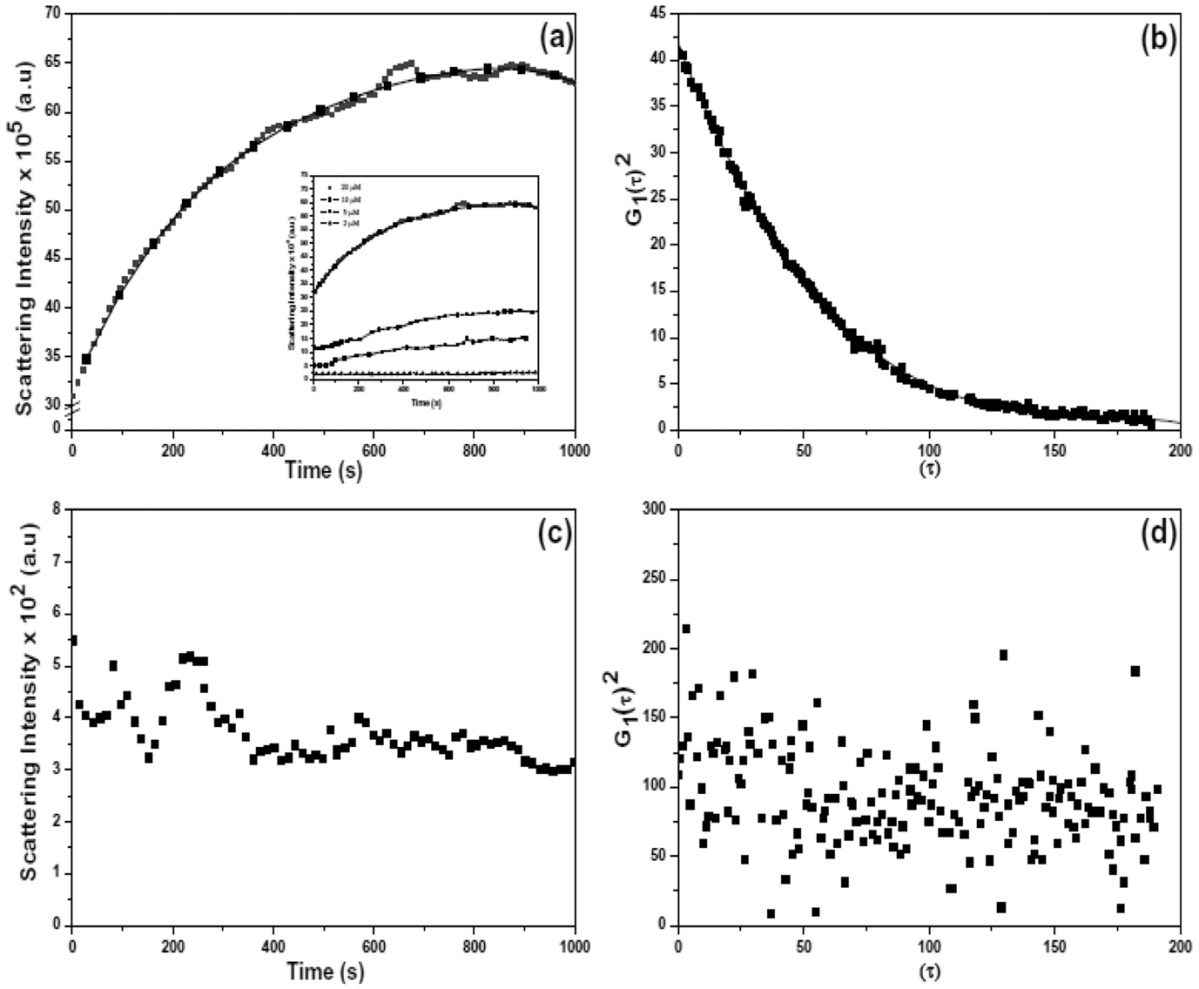
Polymerisation of MamK. (a) Dynamic light scattering of MamK (20 µM) in polymerisation buffer, pH 7.0 at 30°C. The insert shows the concentration dependent polymerisation of MamK under the same conditions (b) recorded net auto-correlation function G^2^(τ) (generated from the reaction of MamK in low salt polymerisation buffer revealing the quality of the second order autocorrelation fit (c) Dynamic light scattering of MamK (20 µM) in high salt polymerisation buffer (100 mM Tris-HCl, 14 mM MgCl_2_, 100 mM NaCl and 30 mM KCl) pH 7.0 at 30°C. (d) recorded net auto-correlation function G^2^(τ) (generated from the reaction of MamK in high salt polymerisation buffer revealing the quality of the second order autocorrelation fit). Polymerisation was initiated by addition of ATP (0.2 mM) and the scattering intensity was measured after a 5 minute incubation period of the assay mixture in the temperature chamber. Light scattering was monitored using a 90° optics scattering angle at a wavelength of 400 nm. Data accumulation period Δτ = 10 µs.

**Figure 2:**
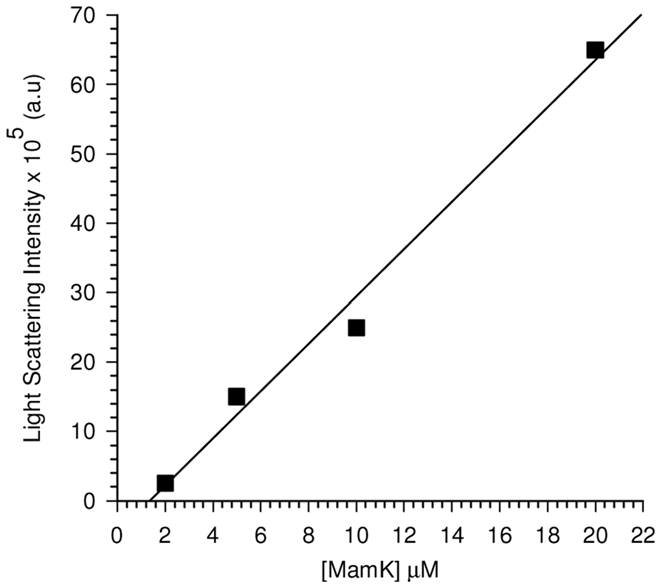
Determination of the critical concentration of MamK. MamK was polymerized in polymerisation buffer in the presence of ATP (0.2 mM) for 1 hr and equilibrated to room temperature. The scattering light intensity was measured for a series of concentrations of MamK. The critical concentration extrapolated from the linear plot was determined to 1.4 µM.

ATP induced polymerisation of MamK was monitored as time**-**dependent increases in light scattering intensity (*I*) detected using 90° optics measured over a period of 1000 s. Multiplication in the intensity of scattered light of MamK after initiation with ATP is exemplified by Figure 1a and typically reflects the nucleation and elongation events of filaments (Equation S1) until a state of equilibrium [23] is reached where the rate of assembly approximately equates to the rate of disassembly depicted by the plateau region. The self**-**association of the protein was shown to occur in P.B at pH 7.0 at 30°C in a concentration dependent manner over a range of 2–20 µM (Figure 1a insert). The rate of polymerisation was too high to observe the lag phase under these conditions, which is also a characteristic of actin**-**like proteins [24], [25] and its absence may have been due to the high concentrations of protein used [5]. The intensity correlation function G^2^[τ] of scattered light from the dynamic activity of MamK over time in P.B is shown in Figure 1b and was analysed by least squares fitting to the multi**-**exponential correlation field. The presence of high salt (100 mM NaCl or KCl) in P.B was shown to be predominantly inhibitory to polymerisation resulting in a random pattern of assembly of a much lower order of atleast 150**-**fold (Figure 1c) when compared to the low salt profile. This effect was accompanied by the loss of the multiphase**-**phase autocorrelation function G^2^[τ] (Figure 1d) suggesting randomisation and a major disruption of the polymerisation process. The decreased conformational stability of MamK in high salt was further reflected by a substantial decline in the melting temperature (T_m_) profile from 66°C (in low salt) to 46°C (in high salt) (results not shown). For G-actin, the value of T_m_ was determined to be 57°C (results not shown). The light scattering intensity was too small to measure at low concentrations of protein and certainly below the critical concentration (1.4 µM) (Figure 2). Therefore, measurements below the critical concentration could not be detected using our instrumentation.

### Assembly Properties of MamK: *In vitro* Polymerisation of MamK Reveals Strong Dependency on Physicochemical Conditions

Parameters affecting the polymerisation of MamK (concentration, salt, divalent metal ions, pH and temperature) are summarised in Table 1. To determine the optimal working salt concentration, the effect of varying KCl in the range of 0**–**200 mM was investigated by monitoring the light scattering intensity of MamK. Reduction in the amount of salt in the P.B was marked by increases in the scattering signal in a concentration dependent fashion consistent with an unambiguous inverse relationship. The maximal polymerisation efficiency achieved in the complete absence of KCl was halved by increasing the salt concentration moderately from 0 to 20 mM. Increasing KCl concentration to a maximum of 200 mM was seen to drastically reduce the polymerisation rate of MamK. The protein also showed a concentration dependent requirement for calcium and magnesium ions in the range of 0.125–2.0 mM. The metal has a primary role in stabilising the bound nucleotide once the ion is tightly bound within the metal binding cavity of actin. MamK showed a similar requirement for divalent cations demonstrating optimal scattering activity in the range of 0.5–1 mM and minimum activity with 2 mM ion concentration showing a 6**-**fold (1.2×10^5^ to 1.9×10^4^) difference between the two concentrations in the assay. Concentrations of Mg^2+^ and Ca^2+^ higher than 1 mM appeared to have an inhibitory effect on MamK. This was in contrast to that reported for MreB from *B. subtilis* [21] where the demand for divalent ions increased with increasing concentration. An earlier study on *thermotoga maritima* MreB however, showed that the rate of polymerisation was marginally higher in the absence of Mg^2+^ and Ca^2+^ at 65°C in 100 mM NaCl and the addition of either 1 mM or 4 mM ion was observed to slow down the assembly rate [15]. This anomaly in behaviour remains to be explained but may be related to temperature**-**induced effects.

**Table 1:** Physico-chemical parameters influencing the polymerisation of MamK.

Parameter Influencing Polymerisation	RangeInvestigated	Polymerisation [Scattering Signal]	
		*Maximum*	*Minimum*
***Divalent cations***			
Calcium ions [CaCl_2_]	0.125–2 mM	1 mM	2 mM
Magnesium ions [MgCl_2_]	0.125–2 mM	1 mM	2 mM
***Salt***			
Potassium Chloride [KCl]	0–200 mM	0 mM	200 mM
***pH***	5.5–9.0	5.5	8.5
***Temperature***	30–45°C	45°C	30°C

The dependence of polymerisation activity of MamK on divalent metal ion concentration, salt, pH and temperature was monitored by light scattering intensity using a 90° optics scattering angle at a wavelength of 400 nm over a period of 1000 s. All polymerisation reactions were performed using a final concentration of MamK of 10 µM in the assay at 30°C. (a) Divalent metal dependent reactions of MamK were performed in 10 mM imidazole at pH 8.0 and initiated by adding 200 µM ATP containing either calcium or magnesium chloride in the range 0.125–2 mM (b) Salt dependent reactions were performed in 10 mM imidazole at pH 8.0 and initiated by adding 200 µM ATP with varying concentrations of KCl in the range 0–200 mM (100 mM, 50 mM, 25 mM, 12.5 mM, 6.25 mM, 3.125 mM, 1.56 mM) (c) pH dependence of MamK polymerization was performed in 0.1 mM EGTA, 1 mM MgCl2, 20 mM KCl, 200 µM ATP with 10 mM MES at pH 5.5, 10 mM imidazole pH 6.5, 10 mM imidazole pH 7.0, 10 mM imidazole pH 7.0, 10 mM tris pH 7.5, 10 mM tris pH 8.0, 10 mM tris pH 8.5, 10 mM tris pH 9.0 and 10 mM tris pH 9.5 (d) Temperature dependence of MamK polymerization was performed in 10 mM imidazole, 0.1 mM EGTA, 1 mM MgCl2, 20 mM KCl, 200 µM ATP in the range 30–45°C.

A quantitative study also revealed accelerating effects of buffer constituents on the assembly rate of MamK other than divalent metal ions. The scattering intensity of MamK varied dramatically in response to pH. In the pH range investigated from 5.5–8.5, increasing the acidity of the assay buffer resulted in increased polymerisation in the presence of 1 mM Mg^2+^ and 0.2 mM ATP. The scattering intensity increased with decreasing pH in an inverse manner reflecting the cross**-**linking activity. This dependency showed that the scattering intensity of MamK at pH 5.5 in P.B (10 mM imidazole replaced by 10 mM MES) was 38**-**fold (7.5×10^4^/2.0×10^3^) higher than at pH 8.5 in P.B (10 mM imidazole replaced by 10 mM Tris) at both extremities. This demonstrated the sensitivity of the protein surface of MamK to specific ions in the medium and their ability to induce self**-**assembly of protein filaments. The pH dependence of MreB was also reported to show a similar trend with maximum polymerisation activity at pH 5.5 which remained unaffected even in the presence of 100 mM KCl [16]. In contrast, the scattering intensity of MamK increased consistently with increasing temperature in the range of 30–45°C.

### MamK is an Effective ATPase and GTPase

#### ATPase and GTPase activity

**Figure 3:**
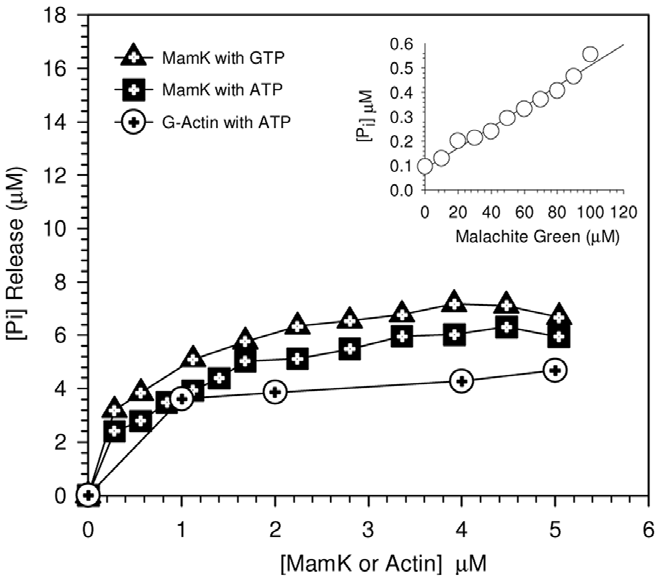
Comparison of ATPase and GTPase activity of MamK with G-actin monitored using a phosphate release assay. Polymerization was initiated by addition of nucleotide at a concentration of 0.2 mM and the (a) hydrolysis for ATP and GTP was monitored by measuring the release of inorganic phosphate, (P_i_) at 630 nm at 30°C as function of concentration. For MamK, the liberation of P_i_ was followed in polymerisation buffer and in G-actin buffer for G-actin. The insert shows the P_i_ -malachite green complex formed in the absence of protein under the same conditions for the rates observed in the presence of protein and were corrected for using the malachite-green standard curve.

The release of inorganic phosphate [P_i_] measured at fixed time intervals (Figure 3) showed that both ATP and GTP are accommodated within the nucleotide binding site of MamK and hydrolysed almost indiscriminately at similar rates which is comparable with actin itself. Hydrolysis occurred rapidly within the first minute implying that both ATP and GTP are instantaneously complexed in the protein**-**nucleotide cavity and that the nucleotide binding domain of MamK is readily accessible to ATP and GTP. The critical concentration of MamK was determined to be 1.4 µM (Figure 2). The absorbance change corresponding to the P_i_ released in the absence of MamK and actin or in the absence of nucleotide was negligible during the time interval of the assay. We also noted a drastic reduction in NTPase activity when pre-heated samples of MamK (100°C) were assayed under the same conditions. This demonstrated that increases in P_i_ were a result of specific interactions with His-MamK and NTPs.

### Size Dependent Dynamic Light Scattering: MamK Preferentially Forms an Assembly of Filaments *in vitro* Similar to Actin which Increases in Size with Temperature

**Figure 4:**
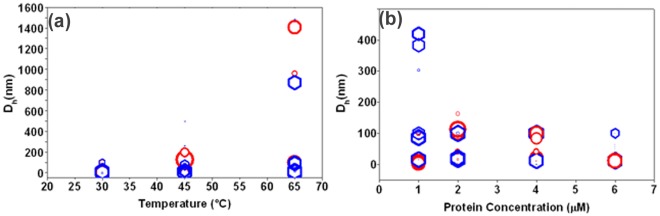
Variability in the size distribution of actin and MamK filaments. Hydrodynamic diameters of MamK (red circles) and actin (blue hexagonals) were measured as a (a) function of concentration (at 1, 2, 4 and 6 µM) and (b) temperature (at 30, 45 and 65°C). Bubble diameters are proportional to the percentage of particles (weighted by intensity) with the respective D_h_ values. The polydispersity index (PDI) that measures the distribution of molecular mass calculated as the ratio weight average molecular weight to the number average molecular weight <1.0.

Figure 4a and 4b shows the variation in the size distribution of MamK and actin filaments from dynamic light scattering (DLS) studies in the presence of Mg^2+^ ions and ATP. The graphs show bubble plots that represent the hydrodynamic diameters (D_h_) of MamK (red circles) and actin (blue hexagonals) as a function of concentration (Figure 4a) and temperature (Figure 4b) at 30°C derived from their diffusional properties (Equation S1). Bubble plots were used to show a comparison of the protein filaments between actin and MamK in terms of D_h_. In addition, the size of the bubble is proportional to the intensity weighted fraction of protein filament assembly with respect to their corresponding D_h_ and therefore provides information about the size distribution of the filaments. In the concentration range from 1–6 µM for MamK, D_h_ averages from 2–16 nm for 90–99% of the filaments formed. Like MamK, the assembly of actin filaments resulted in a similar size distribution of polymers of 2–21 nm between 2–6 µM (90–99%). The size distribution observed from 30 to 45°C was much lower compared to the broad range of filament assemblies seen at 65°C. Temperature dependent increases in D_h_ were observed for both MamK and actin in the range of 11–13 fold (0.17–0.2 µm) and as high as 96**-**fold (1.4 µm) for MamK and 5–35 fold (0.26–0.50 µm) and 62–106 fold (0.87–1.48 µm) for actin at 45°C and 65°C respectively. Hence, highly significant differences are seen as bundling of filaments occurs from nm to µm growth although the dominant populations of filaments exist as 10–35 nm sized particles. This is reflected by the polydispersity index (PDI) which is close to unity (PDI ≤1) and calculated from the ratio of ‘weight average’ to the ‘number average’ of molecular weight and indicates that the vast majority of filaments show a uniform size distribution.

### TEM of MamK Filament Assembly: MamK Shows Filament Bundling when Incubated Irreversibly with a Non-hydrolysable ATP Analogue, ATγP

**Figure 5:**
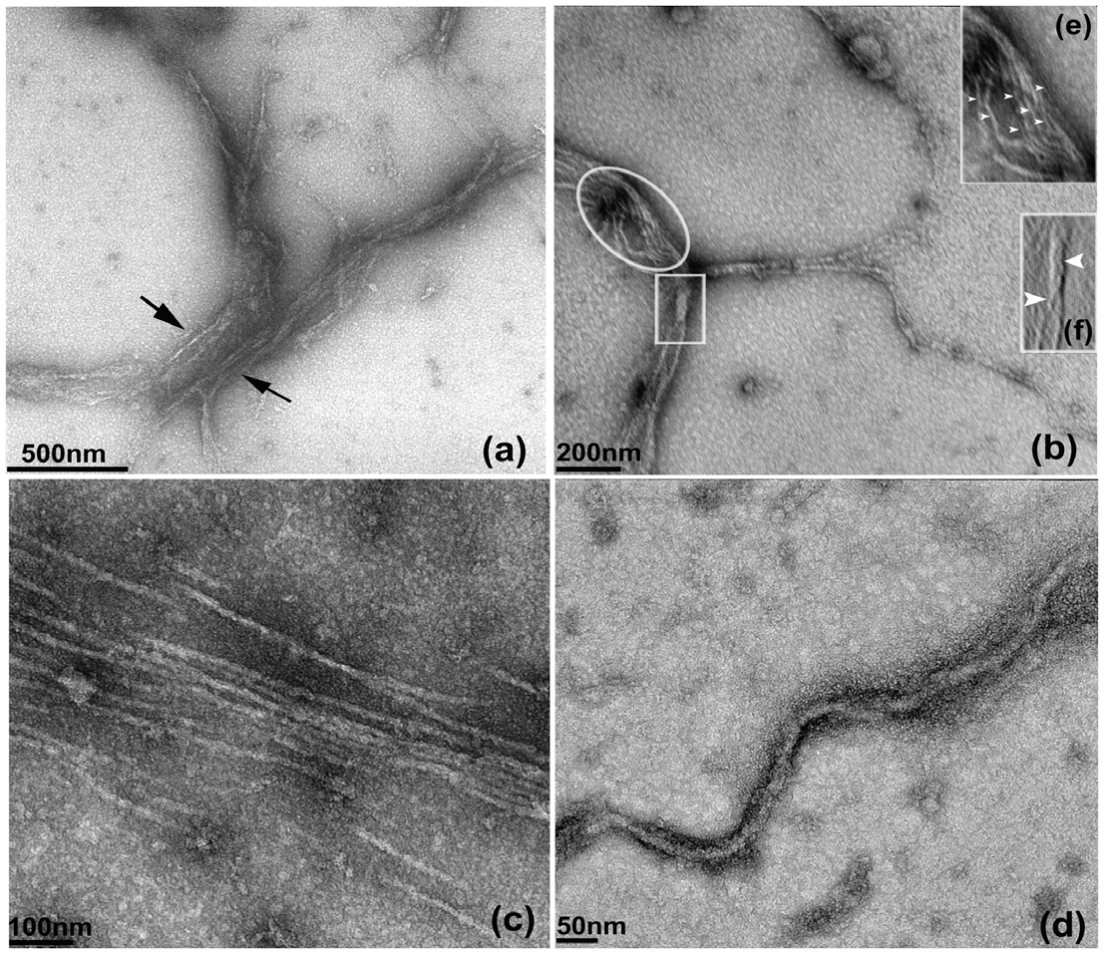
TEM images of MamK filaments. MamK was negatively stained with 2% uranyl acetate and incubated with 0.2 mM ATγP. Incubation of filaments with the nucleotide analogue generated filaments of various sizes (Figure 5a–d). A parallel arrangement of filaments of (∼11–18 nm) was observed after 15 min of incubation (Figure 5c); filament bundling of the order ∼80–270 nm s (Figure 5b). Image 5b also showed the alignment of numerous filaments (circled) in the process of forming bundles composed of smaller filaments of ∼4–12 nm (enlarged; Figure 5e inset) and twisted rope-like morphologies of width ∼38 nm (square) composed of smaller filaments of ∼12–16 nm; (enlarged; Figure 5f inset); a network of individual filaments (16–18) nm in the process of forming bundles of 50–100 nm (Figure 5a) and longer filaments (25–30 nm) of length >2.0 µm (Figure 5d). Figures 5a, 5b and 5d were imaged after 30 mins.

TEM images of MamK shown in Figure 5 were obtained by incubation of the protein with a nucleotide analogue (ATγP) which is more resistant to hydrolysis than ATP. This prevented spontaneous disassembly of filaments which likely arises from the instability of the ADP**-**MamK complex as observed for actin [26], [27]. It has been noted that actin forms narrow microfilaments in the range of 3–6 nm in diameter, an event that rarely occurs since the protein preferentially assembles to form a larger network of bundles. The *in vitro* experiments show that polymerisation activity over longer periods leads to polymer bundling. TEM images suggest that the assembly and disassembly process of MamK results in cytoskeletal structures of varying size both in length and width. The images also show filament size distributions with variable diameters ranging from 11–18 nm (Figure 5c), 25–30 nm (Figure 5d), bundling of filaments of the order 50–120 nm (Figure 5a) from the assembly of smaller filaments of 16–18 and 4–12 nm (Figure 5b). Filament lengths were also observed to be considerably longer and likely reaching lengths in the µm range (Figure 5d and 5a). Filament bundles of this magnitude are proportionally larger in width and size than those observed *in vivo* and may in part be attributed to the effect of ATγP which is less susceptible to hydrolysis then ATP, thereby delaying depolymerisation of the polymers into their monomeric form. However, the use of ATγP has provided a deeper insight of the assembly process by revealing how a number of adjacently localised filaments (4–12 nm) align to form larger bundles and their ability to interact with each other to form ‘twisted rope-like’ structures (Figure 5b inset).

### Structural Homology and Surface Electrostatic Modelling of MamK: Homology Modelling of MamK Reveals a Classical Actin-like Architecture

**Figure 6:**
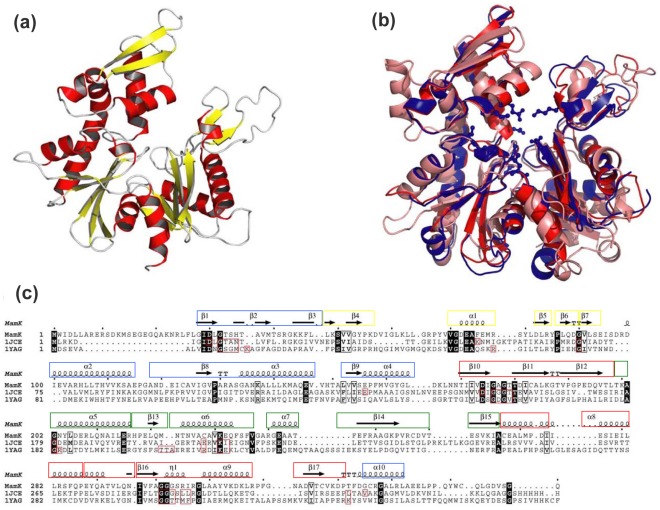
Comparison of the three dimensional and secondary structures of actin and MreB with the predicted structure of MamK. (a) Cartoon representation of the model generated for MamK. (b) Structural superposition of MamK (red), MreB (blue; pdb entry: 1jce) and actin-F (salmon; pdb code 1yag). Those residues involved in nucleotide binding are shown as sticks and balls for MreB. (c) Secondary structural alignment for the three proteins with the active-site residues marked with red boxes as well as the conserved residues shaded in black boxes. The secondary structural elements (β-strands and α-helices) that correspond to conserved domain regions are boxed in blue and red for subdomains DLI and DLII and the variable regions are boxed in green and yellow corresponding to subdomains DSI and DSII.

To correlate the biochemical behaviour with structural characteristics, computational modelling was used to construct a 3**-**dimensional model of MamK using the crystallographic structures of F**-**actin and MreB as templates. The homology generated structure (Figure 6a) showed MamK to retain the overall characteristics of the cytoskeleton fold based on functionally equivalent residues and conserved secondary structural elements. A similar model has been presented by others in an earlier work [21]. As expected for members of the actin family of proteins, the structure can be divided into four sub**-**domains comprising of larger domains shown as DLI and DLII and two smaller ones shown as DSI and DSII. The superimposition of MamK (red) with the crystal structures of F-actin [salmon] and MreB (blue) shown in Figure 6b emphasises the remarkable structural resemblance shared by these proteins. Upon a closer examination of DLI and DLII in MamK, the structural model show secondary structural motifs common to MreB [6] and actin [28], [29]. These regions shown in the sequence alignments of the three proteins in Figure 6c are likely to exist as part of the more conserved domain in DLI (regions boxed in blue) and DLII (regions boxed in red) accommodating the nucleotide binding pocket within the inter**-**domain cleft. The fold of MamK in these domains is seen to conform to the actin**-**like fold composed of the familiar five β**-**strand arrangement surrounded by three α-helices. Experimental evidence to support an actin-type architecture was provided by circular dichroism (CD) studies. The CD spectrum of MamK followed an elliptical pattern strongly resembling G-actin. This was characterised by a pronounced contribution from α-helices signified by a minima around 220 nm in the far-UV region and a positive elliptical turn occurring near 210 nm. The α-helical (*f*_H_) content of MamK determined from its temperature-dependent denaturation profile using the equation *f*_H_ = [(θ_222_–3000)/(−36000−3000)] where θ_222_ is the mean molar residual ellipcity at 222 nm (Figure S2) was determined to be 44% and this value was in excellent agreement with the theoretical prediction of 42%. The value did not significantly differ from the experimentally determined value for G-actin and from those reported previously [30]. Furthermore, a thermal stability study of MamK using CD showed a reduction in the α-helical content with increasing temperature.

The model also predicts the occurrence of helical turns (α2–α4 and α10) and parallel sheets (β10– β12 and β16–17) in MamK which appear at equivalent positions in all three proteins. The variability in the secondary structural elements (boxed in green and yellow) are more obvious around DSI and DSII and this structural divergence may be further reflected by their individualistic cellular functions. Similarly, the structural dissimilarity surrounding some interactions in the model might in part explain the absence of twisted-pair filament morphologies observed for actin [31], [32] and ParM [3]. However, no evidence of such pairing has been observed with MreB. The structural interpretations concerning the inter-domain interactions in MamK are more difficult to predict with certainty in the absence of experimental support and is the subject of on-going studies.

**Figure 7:**
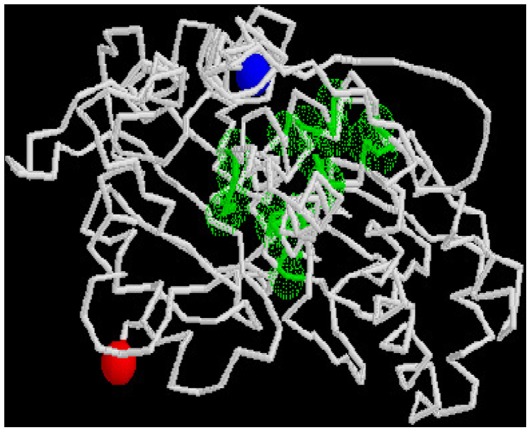
Predicted binding site of MamK. GLY :32(1.29) THR :33(1.29) SER :34(1.29) HIS :35(1.29) GLU :156(0.52) GLY :176(1.29) ALA :177(1.29) THR :179(1.03) GLY :202(1.29) ASN :203(0.80) CYS :228(1.29) LYS :231(1.29) GLU :232(1.29) SER :235(0.74) GLY :301(1.04) GLY :302(1.29) GLY :303(1.29) ARG :305(1.29) ILE :306(1.29) THR :332(1.04). Binding site residues reported above are derived from a consensus prediction of the top 10 functional homologs, which are selected on the basis of structural alignment between the predicted model and the PDB structures. Values shown inside the parenthesis beside each residue reflect the relative frequency with which the predicted residue in the model structurally aligns with known binding site residue in the functional homolog(s). A relative frequency >1 signify a binding site residue prediction with high confidence and vice-versa. Predicted binding site residues are shown in green sphere while N and C terminus in the model are marked by blue and red spheres respectively.

The cavity that accommodates nucleotides and divalent cations is depicted in MreB as sticks and balls protruding from the base of the cleft between domains DLI and DLII. The active site and the highly conserved residues for actin and MreB are shown as red and black boxes respectively in the sequence alignment pattern (Figure 6c). A comparison suggests that the nucleotide binding domain of MamK may share the same nucleotide binding specificity given the high structural similarity particularly with MreB. To determine the relative potential of binding sites in MamK, a consensus sequence prediction was applied against known functional homologs within the predicted binding site (Figure 7). Inspection of the 20 predicted residues showed that seven of the residues are present at the same positions (35%) in all three proteins, 9 are present only in MamK (45%), 3 are shared with MreB (15%), and only one with actin (5%) at the same positions. Hence, most of these binding residues are unique to MamK of which 2 (22%) reside in DLI, 4 (44%) in DLII and 3 (33%) in DSII but none in DSI.

**Figure 8:**
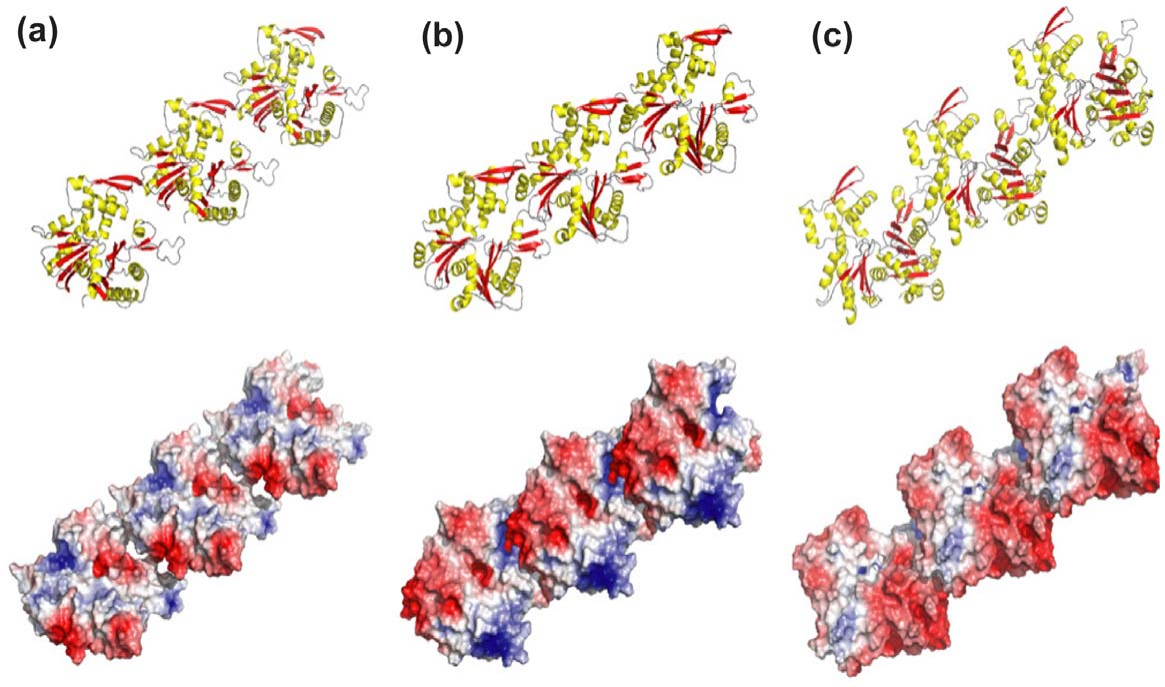
3-dimensional filament modeled structures of the fiber-like structures of MamK, MreB and actin-F. 3-dimensional modeled structures of the fiber-like structures of MamK (A), MreB (B) and actin-F (C) as they would form filaments are shown in the upper panel and the corresponding global electrostatic surface potentials of each protein contoured at 5 kT are shown in the lower panel. The active site of MreB is indicated by the arrow and is likely to reside in a similar position in MamK and actin. Red represents regions of negative electrostatics, blue represents regions of positive electrostatics, while white is neutral. This analysis was carried out using Adaptive Poisson-Boltzmann Solver (APBS) through solution of the Poisson-Boltzmann equation (PBE) using default parameters with contour values ranging from −5 to 5.

The low sequence identity shared by MamK, MreB and actin signifies large regions of non**-**conserved residues that increase the possibility of functional variation in protein**-**protein interactions. These specific interactions are not only dictated by the molecular shape of proteins but also charge**-**charge electrostatic associations at the binding interface. The importance of this is illustrated by Figure 8 which shows the modelled 3**-**dimensional structures of (a) MamK, (b) MreB and (c) actin as they would assemble into filaments (upper panel). The orientation and shape complementary is preserved in the corresponding global surface electrostatic maps (lower panel) highlighting differences in protein clusters marked by positive (blue), negative (red) and neutral (white) electrostatic regions; such electrostatic fields are systemic properties and are not revealed by simple structural comparisons. The variation in the surface pattern in relation to the charge distribution is observed to be significantly different among all three proteins. The divergence in sequence generates marked differences in the electrostatics between them and consequently different plausible polymerization driving forces and mechanical properties. These variations are not surprising given the obvious differences emphasised by sequence comparisons and topological orientations. The charge distribution is also observed to follow a unique ‘periodic’ pattern in each protein revealing strong geometric complimentary between the negatively charged carboxylate and positively charged basic groups and neutral lobes. The surface shape configurations between the proteins are distinctly dissimilar resulting from localised fluctuations in electrostatic charges etched as low and high regions. The periodic depressions that occur along the surface are particularly pronounced in actin when compared to MamK and MreB. The molecular surface of MamK appears to be largely hydrophobic or neutral. In contrast, actin is characterized by a large anionic surface. Comparison of MreB with MamK on the other hand demonstrates a relatively closer electrostatic and shape complementary (−6) which in part may explain their similar biochemical behaviour.

## Discussion

The results of this study show MamK to be archetypical in its overall topology and behaviour to the actin**-**like super family of proteins despite its unique origin and peculiar role as a bacterial magnetotactic protein. The findings unequivocally support the suggestion that MamK may indeed be a product of a common ancestral gene. The low sequence identity shared among its members is not correlated by the same degree of structural divergence (as suggested by structural modelling [33], [34] based on homology with actin), suggesting greater conservation. Indeed, despite the limited resolutions of such models, they nevertheless have proved to be immensely useful in rationalizing to some extent the observed physicochemical properties.

Functionally diverse actin-like proteins share a common ATPase domain [35] and ATPase activity is involved in regulating the polymerisation process. However, the observation that metal bound MamK is seen to spontaneously polymerise in the absence of ATP as previously observed [20], [21] questions the absolute requirement of nucleotides to aid the assembly process. There are however, suggestions that some cytoskeleton proteins form disordered morphologies in the absence of ATP or GTP as reported for MreB [36]. Early studies demonstrated that Ca^2+^ stabilises the G**-**actin complex even in the absence of ATP [37]. However, impairment of the metal coordinating residue in ParM by mutagenesis showed that the polymerisation activity remained unhindered in the presence of ATP and the dynamics of filament disassembly was largely reduced in the absence of Mg^2+^ ions [38]. This observation together with those seen for the MamK protein signifies that the hydrolysis of ATP may primarily relate to its role in the dissociation of aggregated filaments but not in the polymerisation process itself.

The autocorrelation function G^2^[τ] generated from the scattered intensity during polymerisation of MamK in the presence of divalent metals and ATP is observed to follow a multi**-**exponential decay. This pattern corresponds to the assembly and disassembly of filaments that co**-**exist as monomeric, oligomeric and polymeric sized filaments. The derivatized exponential profile of MamK during polymerisation showed an initial rapid decay corresponding to the diffusion dynamics of monomeric MamK followed by a slower phase as polymerisation progressed from nucleation to a higher ordered state. Given that a mono**-**dispersed sample adheres to a single exponential decay, the multi**-**exponential dynamics observed for MamK during light scattering experiments suggests a degree of poly**-**dispersity during polymerization. The variation in D_h_ for both actin and MamK indicate that filaments have a tendency to form higher ordered filaments under physicochemical conditions that favour polymerisation. Clusters composed of hundreds of filaments have been reported for actin [31], [39] and in exceptional cases, very large bundles of actin ranging from 3.5–9.1 µm were also evidenced from light scattering experiments [40]. Typically, as suggested by the PDI and small angle scattering experiments, MamK filaments exist as small but multi-monomeric cylindrical structures composed of 2–6 monomers (results not shown). This is consistent with a typical actin polymer composed of a 5–20 nm filament network with total width ≈28 nm [32]. The diffusion properties of MamK observed from D_h_ light scattering experiments signify a behavioral pattern that deviates from a non**-**spherical or monomeric arrangement. The temperature induced pattern that leads to broader variability in the mass distribution of MamK might suggest conformational changes that favour increased bridging among filaments. Calorimetric investigations have shown that ATP may play a role in stabilising actin preventing the disintegration of the protein at higher temperatures [41] and this increased stabilisation is afforded by the release of P_i_ from the bound F-ADP complex [42].

In this study, it has been observed that polymerisation of MamK seems to occur most efficiently at fairly low ionic concentrations (0–20 mM KCl) and is substantially inhibited at ionic strengths that are generally thought to exist within the cell environment (KCl∼100 mM) [43]. This may also be underscored by the strong electrostatics that characterize their surfaces as is apparent from our structural models. This functional irregularity is also observed for MreB [16] however, it does not prevent assembly of the protein *in vivo* [11], [12]. The low salt requirement may be physiologically relevant in limiting the spontaneous assembly of MamK to a level that is sufficiently regulated in a controlled and dynamic fashion within the bacterial cell. In contrast to this finding, the optimal conditions that favoured polymerisation of MamK were achieved using a buffer composed of 1 mM Mg^2+^/Ca^2+^, pH 5.5 and 30°C temperature for the range investigated in this study.

The variability in size of MamK was supported by TEM imaging and was consistent with those seen for the *in vitro* study of *M. magnetotacticum* strain of MamK [20]. Filament bundles were also visualised *in vivo* in AMB**-**1 using cryo**-**electron tomography [11] and in MSR-1 cells [44]. These filaments are usually characterised as long straight structures and join together to form compact thick bundles. In the presence of salt and neutral pH, MreB was also observed to form filaments of varying sizes and shown to exist either as straight, arched or coiled circular structures with a width size between 6–20 nm [6]. The finding that MreB forms sheets of bundles [15], [36] similar to those seen in MamK in contrast to protofilament assembly associated with actin offers a clue about their functional role within the cellular environment. However, recently MreB was also shown to form multilayered sheets in *Thermatoga maritime* [45] that were observed as diagonally interwoven filaments and were several µm in length. MamK shows an inherent ability to form filaments that vary broadly in length and width supporting the results of dynamic light scattering. Interestingly, the use of the ATP analogue ATγP has also provided a snapshot view of the assembly process revealed by TEM studies. In this study, in vitro analysis by TEM and dynamic light scattering showed MamK to preferentially form filaments of the order of 12–18 nm that assemble to form larger filaments. This process involved the systematic alignment of 8–10 filaments adopting a parallel-like arrangement prior to forming adjacently positioned bundles. A closer examination of a section of the TEM image also showed MamK to conform to a ‘twisted rope-like’ arrangement of intertwined filaments. However, it is likely that a detailed and precise microstructural analysis will require more sophisticated methodologies to capture and stabilise these events for a more intricate study. This may necessarily involve developing novel preparatory techniques for imaging less stable proteins such as MamK and in particular for visualising protein assemblies. The TEM images obtained for MamK may be different to the twisted morphologies as in MreB suggesting functional differences may exist between both proteins. This however, remains to be established. Packing of filaments into large clusters affords a considerably larger mechanical thrust and increased rigidity for the filaments to act as a cellular frame to maintain the shape of cells. In the same context, several MamK filaments bundled together provides rigidity to the surrounding magnetosomes maintaining their alignment to aid magnetotaxis [46]. Such an arrangement was indeed revealed by cryo**-**electron microscopy in *Magnetospirillium magneticum* and in MSR-1 showing the alignment of up to at least seven filaments around 6 nm in width providing a supporting backbone to the magnetosomes. In view of the current knowledge of the assembly properties of actin, it is conceivable that MamK under defined conditions is capable of adopting a variety of monomeric and oligomeric morphologies but preferentially may exist in a predominant form inside the cell. Indeed, this is a likely scenario in magnetotactic bacteria in line with the observation that filaments of MamK emerge site specifically and simultaneously in different locations within the cell.

Remarkably, despite the low sequence identity between MamK and MreB, there are similarities and differences between the two proteins in their biochemical behaviour. MamK exhibits almost identical characteristics to MreB in response to salt, temperature and pH effects and its interaction with divalent metal ions [14], [16] and yet differs markedly from the salt**-**induced behaviour of actin in the presence of ATP [41]. This finding reiterates the hypothesis that proteins with low sequence identity can adopt similar structural folds with contrasting functionality within the same protein family. It is also noteworthy that despite the similarity in the structural fold among members of the same protein family, function can be modulated by varying systemic properties such as electrostatic surface characteristics [47] as observed here. The experimental structure of MamK will no doubt reveal subtleties that elude our investigations in this study. Nevertheless, homology models and electrostatic analyses have provided correlations with some of the biophysical characteristics of MamK, MreB and actin and a basis to speculate on differences in behaviour.

## Materials and Methods

### Sub-Cloning, Protein Expression and Purification of MamK

The *mamK* gene from *M. gryphiswaldense MSR-1* (GenBank: CAE12034.1) was obtained as a MamK-pGEM construct (10 µg) and digested from the construct at restriction sites SalI/EcoRI. The digested product corresponding to the *mamK* gene (1083 bp) was purified, amplified (116 ng/µl) and sub-cloned into a pET29b protein expression vector (Novagen). The MamK-pET29b protein expression vector was transformed in *E. coli* C41(DE3) cells (Lucigen) and cultures were grown at 37°C in auto inducible media ([5052 ZYM; [48] supplemented with 30 µg/ml kanomycin and grown for 24 h. Cells were harvested and the C-terminal poly-histidine-tagged MamK was purified as a soluble protein in accordance with procedure described in Protino®**-**Ni-TED/IDA (Marcherey**-**Nagel) protocol using varying concentrations of imidazole as elutent. To ensure the complete removal of contaminating proteins, His-MamK purifications corresponding to imidazole fractions 20, 50 and 100 mM were pooled and further purified using a spin column (amicon) with a molecular weight cut-off (MWCO) of 100 KDa. The eluate containing His-MamK was subjected to a second purification step using a spin column (thermoscientific) with a MWCO of 40 KDa. The purified fraction was concentrated and dialysed in polymerisation buffer (P.B) (10 mM imidazole, 1 mM MgCl_2_, 0.1 mM EGTA, 20 mM KCl, pH 7.0). Prior to use, protein fractions were centrifuged at 90,000 *g* for 2 h. The protein was purified to homogeneity and purity was assessed using SDS-PAGE.

### Preparation of G-actin

Lyophilised G**-**actin was prepared by solubilising the protein in water until a homogenous solution was observed without fibres. The protein was dialysed against G**-**actin buffer (2 mM Tris, 0.2 mM ATP, 0.2 mM CaCl_2_, 0.005% (w/w) NaN_3_ and 0.2 mM DTT). Prior to use, the protein was centrifuged at 90,000 *g* for 2 h.

### Transmission Electron Microscopy

Purified MamK (1 mg/ml) in P.B was incubated at 30°C for 30 min. Carbon**-**coated grids were deposited with 5 or 10 µl of the reaction mixture and left for filament absorption. The excess sample was absorbed away with the from the grid surface using a filter paper. The grids were immediately washed with 10 µl of water and finally stained with 2% uranyl acetate and allowed to air dry. Transmission electron microscopy investigations were performed on a Zeiss LIBRA 200 EM operated at 200 kV and on a Zeiss EM 912X operated at 120 kV. The images were processed and analysed using Image J ([49]; http://imagej.nih.gov/ij).

### Circular Dichroism

Far**-**UV CD spectra of MamK were obtained using a Jasco spectropolarimeter (Jasco PS150J, Tokyo, Japan) in polymerization buffer at room temperature using a 1 mm path quartz cuvette. In addition, ellipticity data were recorded between 200 and 300 nm as a function of temperature at 5°C intervals up to 90°C with 10 min equilibration periods. Melting profiles were determined by plotting ellipticity at 222 nm against temperature. All profiles were referenced against a baseline correction measured in buffer in the absence of the protein.

### Phosphate Release Assay

ATP dependent activity of MamK and Actin was measured using the malachite green**-**sodium molybdate assay [50] by measuring the release of inorganic phosphate [P_i_] using a modified procedure described previously [15]. Polymerisation reactions were initiated by the addition of ATP (0.2 mM) in polymerisation buffer at 30°C and monitored at 630 nm using a pure grade 96**-**well plate (Brand GmbH, pure grade) using a plate reader (Multi-scan Ascent, Thermo electron corporation). The data was analysed using the Ascent software. The linearity range for the continuous mode measurement was 0**–**3 Abs ±2% with a resolution of 0.001 Abs and 9 s measurement time. The readings were corrected for non**-**specific reactions using a phosphate standard curve in the absence of the protein.

### Dynamic Light Scattering

Size distribution measurements were performed with a multi**-**angle optics DLS instrument (Malvern zetasizer, nano ZS) using a 532 nm ‘green’ laser equipped with a temperature regulator. The instrument incorporates a universal ‘dip’ cell with a narrow band filter. Polymerisation time course light scattering experiments were performed with a 90° optics scattering angle (Malvern zetasizer, 3000 HS) equipped with a temperature regulator and measurements were recorded at a wavelength of 400 nm. The data was analysed using the zeta**-**sizer software (Dispersion Technology, *v4.20*).

### Critical Concentration of MamK

Varying concentrations of MamK were polymerised in P.B in the presence of 1 mM Mg^2+^ and ATP and equilibrated to room temperature for 1 h. Light scattering intensity was measured for each concentration of MamK in the assay and the critical concentration was determined from the linear extrapolation of the concentration *vs* maximum scattering intensity plot.

### Structural Modelling of MamK

The program Modeller (http://salilab.org/modeller/) has been used to construct the 3D structure of MamK, based on the chain A of the rod shape determining protein MreB structure (Protein Data Bank entry: 1jce) and F-actin (pdb entry: 1yag). The alignment used to build such a model was obtained using a profile**-**profile alignment and fold recognition algorithm (FFAS). This method relies on the amplification of the sequence conservation pattern present in sequences of homologous proteins in order to detect remote homologies beyond the reach of some other sequence comparison methods. The sequence identity between the two proteins was 22%. We further used the server Firestar (http://firedb.bioinfo.cnio.es/Php/FireStar.php), to predict functional residues for the sequence of MamK and also I-TASSER server (http://zhang.bioinformatics.ku.edu/I-TASSER) to predict the binding site residues in the MamK protein. This algorithm searches 90% redundant pdb using PSI-BLAST to generate a profile that is further used to search against the template database. In order to examine the possibilities of polymer formation, we constructed a trimer of MamK guided by the crystallographic symmetrical units found in the structure of 1jce and used this to generate a fiber**-**like polymer. Electrostatic analyses of these structures were carried out by solving the linear Poisson**-**Boltzmann equation numerically using the software package Adaptive Poisson**-**Boltzmann Solver (APBS).

## Supporting Information

Figure S1 **Concentration dependent elution of over-expressed MamK with increasing imidazole.** (a) Sodium dodecyl sulfphate-polyacrylamide gel (12%) electrophoresis of purified histidine-tagged MamK eluted using an imidazole gradient to reduce the co-purification of contaminant proteins binding to the column resin. MamK was eluted with increasing concentrations of imidazole in accordance with the procedure described in Protino®-Ni-TED/IDA protocol (Marcherey-Nagel). Figure S1(b) and (c) shows the purified over-expressed His-MamK which migrates with a molecular weight corresponding to ∼42 KDa verified by the molecular weight standards (Page ruler pre-stained protein ladder, Fermentas). The gels were stained with brilliant coomassie blue G-250.
(TIF)

Figure S2 **Thermal stability of MamK and determination of the α-helical content.** Thermal denaturation of MamK (1 mg/ml) was measured by circular dichroism at 222 nm in polymerisation buffer as a function of temperature. The melting temperature of MamK was 66°C. The insert shows the change in the helical content of MamK as a function of temperature. The fractional helicity (f_H_) was calculated using the equation [(θ_222_–3000)/(−36000−3000)] where θ_222_ is the mean molar residual ellipcity at 222 nm and was determined as 44%.
(TIF)

Equation S1 **Correlation function and translation diffusion coefficient expressions of scattered intensity.**
(DOC)
