# Supplementary figures and images for: Insight into the Assembly Properties and Functional Organisation of the Magnetotactic Bacterial Actin-like Homolog, MamK
Source: PLoS One. 2012 May 7;7(5):e34189. doi: 10.1371/journal.pone.0034189 (PMC3346761; doi:10.1371/journal.pone.0034189)

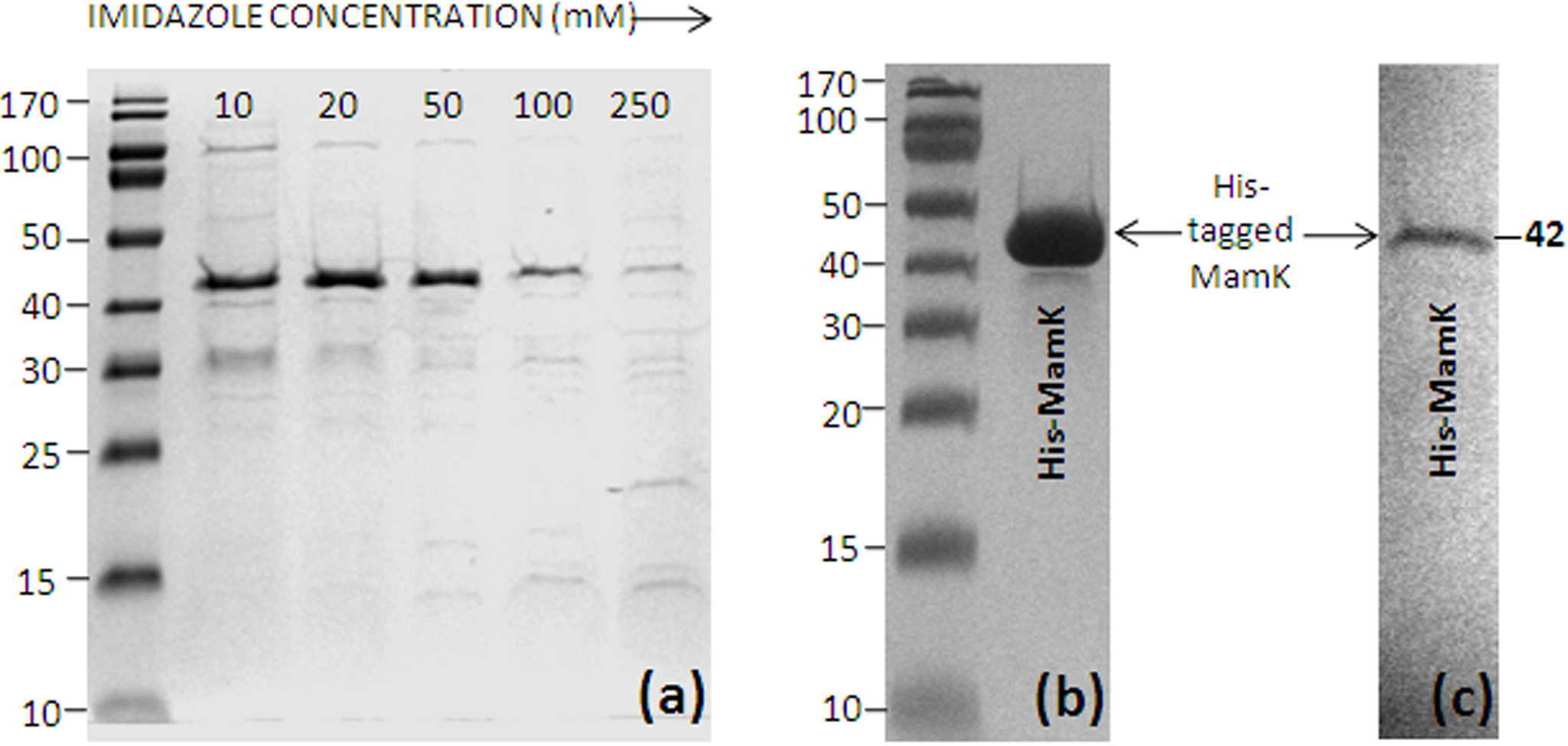

Supplement: Figure S1 — Concentration dependent elution of over-expressed MamK with increasing imidazole. (a) Sodium dodecyl sulfphate-polyacrylamide gel (12%) electrophoresis of purified histidine-tagged MamK eluted using an imidazole gradient to reduce the co-purification of contaminant proteins binding to the column resin. MamK was eluted with increasing concentrations of imidazole in accordance with the procedure described in Protino®-Ni-TED/IDA protocol (Marcherey-Nagel). Figure S1(b) and (c) shows the purified over-expressed His-MamK which migrates with a molecular weight corresponding to ∼42 KDa verified by the molecular weight standards (Page ruler pre-stained protein ladder, Fermentas). The gels were stained with brilliant coomassie blue G-250. (TIF) [file pone.0034189.s001.tif]

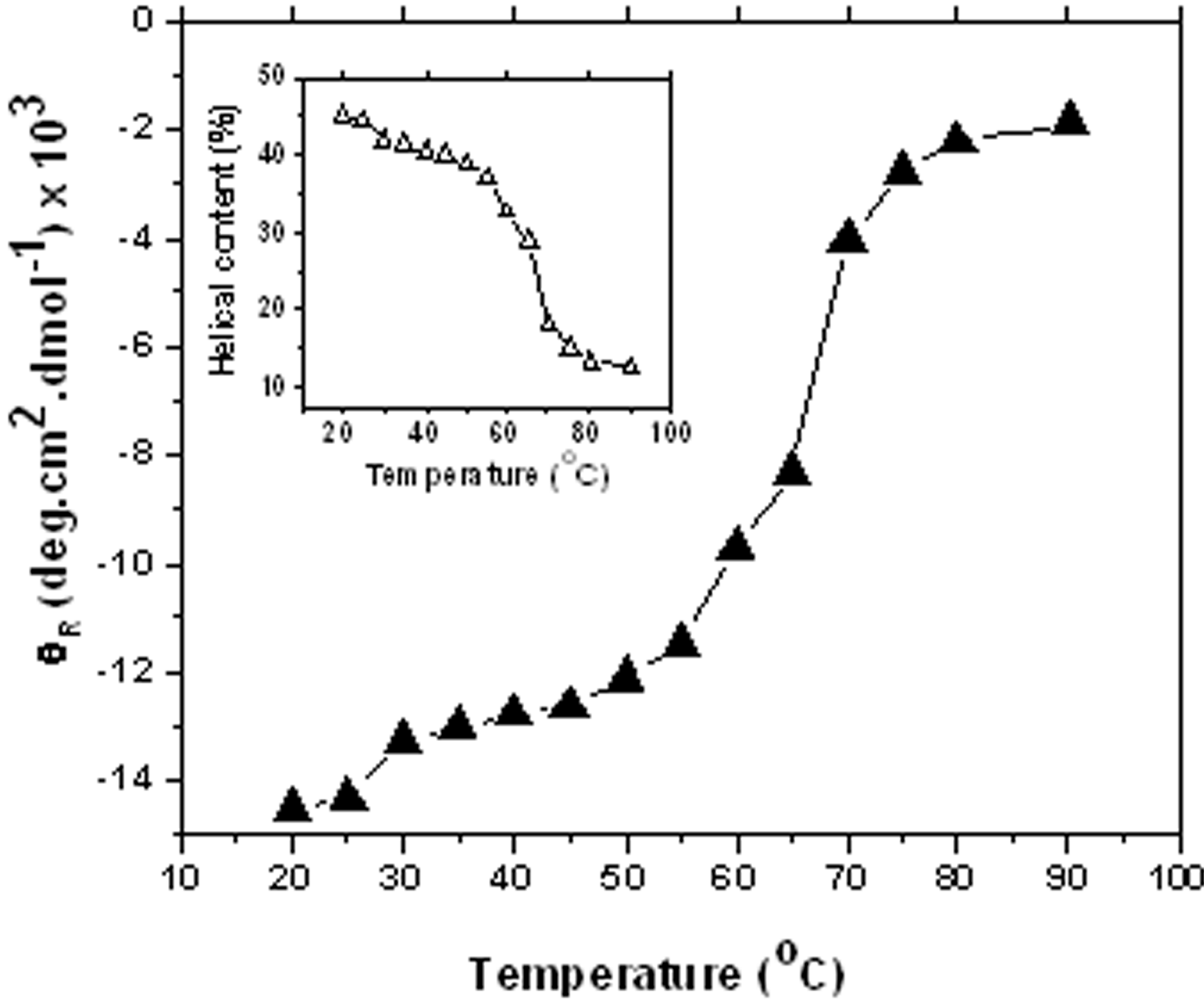

Supplement: Figure S2 — Thermal stability of MamK and determination of the α-helical content. Thermal denaturation of MamK (1 mg/ml) was measured by circular dichroism at 222 nm in polymerisation buffer as a function of temperature. The melting temperature of MamK was 66°C. The insert shows the change in the helical content of MamK as a function of temperature. The fractional helicity (fH) was calculated using the equation [(θ222–3000)/(−36000−3000)] where θ222 is the mean molar residual ellipcity at 222 nm and was determined as 44%. (TIF) [file pone.0034189.s002.tif]
