# Supplementary material for: Insight into the Assembly Properties and Functional Organisation of the Magnetotactic Bacterial Actin-like Homolog, MamK
Source: PLoS One. 2012 May 7;7(5):e34189. doi: 10.1371/journal.pone.0034189 (PMC3346761; doi:10.1371/journal.pone.0034189)
Supplement: Equation S1 — Correlation function and translation diffusion coefficient expressions of scattered intensity. (DOC) [file pone.0034189.s003.doc]

**Equation S1**

**Correlation function and translation diffusion coefficient expressions of scattered intensity**

The movement of particles in suspension can be characterised by a ‘diffusion coefficient’ and the translational diffusion coefficient D_T_ for the Brownian process is ‘normalised’ by the wave vector K to give the relaxation time Γ for the diffusion as

Γ = D_T_K^2^  Equation (1a)

In its most simple form for a globular scatter, the correlation function G^2^(τ) expressed for a first order auto-correlation function which predicts the degree of correlation in the signal as a function of the correlation or lag time, τ

G^2^(τ) = e^-τ /Γ^ Equation (2a)

For correlation functions that deviate from single exponential behaviour, a cumulant expansion of equation (2a) leads to a more useful term given by equation (2b) that can be applied to multi-exponential functions

$G^{1} (\tau)=\sum_{i} A_{i} e^{-\Gamma_{i} \tau}$ Equation (2b)

where and Γ_1_ > Γ_2_ >Γ_3_ > and its integral form is given by equation (2c)

$G^{2} (\tau)=\lim_{T-\infty} \left[\frac{1}{T}\int_{0}^{T} I\left(t\right)I\left(t+\tau\right)dt\right]$ Equation (2c)

where *I* = mean scattering intensity (counts rate /s)

and ( t + τ ) = incremental increase in correlation time or delay time (s^-1^)

Translational diffusion coefficients are related to the hydrodynamic radius of scattering particles through the Stokes-Einstein formula (equation 2d)

Equation (2d)

*R*_h_ = radius of a sphere with the same hydrodynamic diffusion coefficient as the particle in the sample

*k*_b_ = Boltzman’s constant

*T* = temperature (Kelvin) and

η = viscosity of solvent
